# The GPlates Portal: Cloud-Based Interactive 3D Visualization of Global Geophysical and Geological Data in a Web Browser

**DOI:** 10.1371/journal.pone.0150883

**Published:** 2016-03-09

**Authors:** R. Dietmar Müller, Xiaodong Qin, David T. Sandwell, Adriana Dutkiewicz, Simon E. Williams, Nicolas Flament, Stefan Maus, Maria Seton

**Affiliations:** 1 EarthByte Group, School of Geosciences, The University of Sydney, Sydney, NSW 2006, Australia; 2 Scripps Institution of Oceanography, La Jolla, California, 92093, United States of America; 3 CIRES, University of Colorado at Boulder, Boulder, Colorado, 80309, United States of America; Universidade de Vigo, SPAIN

## Abstract

The pace of scientific discovery is being transformed by the availability of ‘big data’ and open access, open source software tools. These innovations open up new avenues for how scientists communicate and share data and ideas with each other and with the general public. Here, we describe our efforts to bring to life our studies of the Earth system, both at present day and through deep geological time. The GPlates Portal (portal.gplates.org) is a gateway to a series of virtual globes based on the Cesium Javascript library. The portal allows fast interactive visualization of global geophysical and geological data sets, draped over digital terrain models. The globes use WebGL for hardware-accelerated graphics and are cross-platform and cross-browser compatible with complete camera control. The globes include a visualization of a high-resolution global digital elevation model and the vertical gradient of the global gravity field, highlighting small-scale seafloor fabric such as abyssal hills, fracture zones and seamounts in unprecedented detail. The portal also features globes portraying seafloor geology and a global data set of marine magnetic anomaly identifications. The portal is specifically designed to visualize models of the Earth through geological time. These space-time globes include tectonic reconstructions of the Earth’s gravity and magnetic fields, and several models of long-wavelength surface dynamic topography through time, including the interactive plotting of vertical motion histories at selected locations. The globes put the on-the-fly visualization of massive data sets at the fingertips of end-users to stimulate teaching and learning and novel avenues of inquiry.

## Introduction

Geoscience relies heavily on the visualization of geospatial data, helping us to make fundamental new inferences about the Earth around us, and how it has evolved. More than 100 years ago, Alfred Wegener [1] proposed the theory of continental drift by establishing previously unrecognised associations between geological and fossil data from widely dispersed continents, and fitting together the bathymetric outlines of these continents defined within maps of the Earth. Today, scientists have vast quantities of data at their disposal to pursue new theories of the Earth system—the Earth’s surface is continuously monitored by satellites, geological sampling from land and sea is being assembled into community databases, geophysical imaging probes present-day deep Earth structure, and supercomputing resources power dynamic simulations of the physical processes shaping the world around us, over timescales up to hundreds of millions of years. These advances also create new problems to solve—how can scientists efficiently share and communicate these data sets with each other, and with non-specialists, to maximise the public benefit of the large efforts undertaken in creating these data sets?

Virtual globes have changed the way we interact with spatial data, largely due to the popularity of Google Earth [2] and NASA World Wind [3]. Following the broad uptake of these technologies by scientists and end-users, alternative technologies for virtual globes have been developed that are faster for displaying of very large data sets (e.g., for detailed 3D textured city models [4]), work well on hand-held devices, and can function entirely within a web-browser. These alternative technologies also provide powerful additional functionality, for instance use of custom imagery and terrain models with greater user-control of vertical exaggeration; a dedicated infrastructure for displaying time-dependent data sets; and the ability to produce custom user-interfaces, specifically designed to showcase the results of a particular study or project. These and other requirements have prompted the development of the Cesium software library (cesiumjs.org).

Here, we describe novel developments in the use of Cesium virtual globes to enable web-based visualization and knowledge discovery for a range of applications in the geosciences. We have created tools that allow end-users to interact with data sets that describe the Earth as it is now, based on remote sensing data, marine geophysical data and a novel approach of mapping seafloor lithology. In addition, our virtual globes allow the user to visualize Earth’s plate tectonic evolution through data-driven reconstructions of supercontinent dispersal and global numerical models describing how mantle convection has shaped the Earth’s surface through time. We discuss the technical details required to implement these tools, and illustrate how they can help to increase public engagement with geoscience, and promote cross-disciplinary research by making geospatial data sets more accessible to a wide audience within the scientific community.

## Technical Background

### System Architecture

Cesium is an open-source JavaScript library built on the Web Graphics Library (WebGL) for interactive visualization of 3D globes and 2D maps in a web browser. WebGL is a web standard, designed and maintained by the Khronos Group (www.khronos.org), for low-level 3D graphics Application Programming Interface (API) based on OpenGL ES 2.0 –a standard API for 2D and 3D graphics on embedded systems [5]. The web browsers implement WebGL API as a Document Object Model (DOM) interface in HTML5 [6] Canvas elements, which allows JavaScript programs to utilize Graphics Processing Unit (GPU) for 3D graphics hardware acceleration. WebGL support is widely available in modern browsers, such as Google Chrome, Mozilla Firefox, Safari, and others. As a virtual globe and map engine, Cesium utilizes a WebGL API to provide fast, cross-platform, cross-browser and plugin-free 3D rendering functionality, which makes it ideal for the development of interactive 3D global geophysical and geological data visualization web applications.

**Fig 1:**
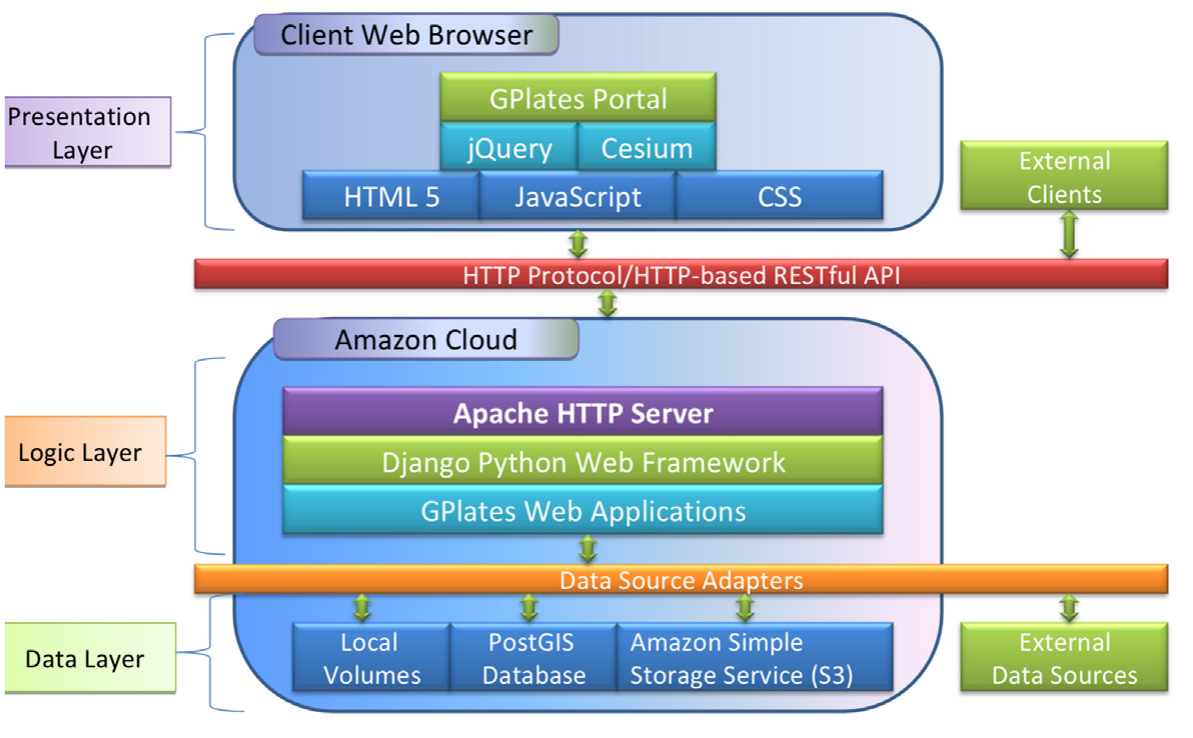
The GPlates Portal’s multilayered architecture. A three-layered architecture consists of presentation, logic and data layers. The presentation layer includes the Graphical User Interface (GUI) and the main canvas for 3D rendering. The logic layer contains a number of web services written in Python and deployed in Django Web Framework [7]. The data layer focuses on the storage and retrieval of data.

The GPlates Portal (portal.gplates.org) is designed with a three-layered architecture consisting of presentation, logic and data layers. The layer is a logical concept of component groups, which divides a large system into different areas of functionality so that the complexity of the system can be minimized and each layer can be designed and implemented separately and efficiently. This multilayer architecture also provides the highest level of encapsulation and isolation, which decouples the system and reduces the ripple effect of changes to the system. Fig 1 illustrates the multilayered system architecture of the GPlates Portal.

The presentation layer includes the Graphical User Interface (GUI) and the main canvas for 3D rendering. The GUI is developed with HTML5, JavaScript and Cascade Style Sheet (CSS) and can be used to control the main canvas and display additional information. The jQuery JavaScript library is used to help with the client-side scripting and the Cesium library is used to draw the 3D virtual globe and maps on an HTML5 Canvas.

The logic layer contains a number of web services written in Python and deployed in Django Web Framework [7]. The Apache HTTP Server is used to handle the HTTP protocol between the Django Web Framework and clients. The HTTP requests are dispatched to corresponding web services by the Django Web Framework and the HTTP responses are delivered back to clients through the same stack.

The data layer focuses on the storage and retrieval of data. A set of data source adapters is created to decouple the web services from the data storage details. The data source adapters read a Data Source Description (DSD) file in JSON format to retrieve and feed data back to web services. The web services only interact with an abstract interface of data source adapters, so any changes to the data storage are completely transparent to the web service. This decoupling allows us to change the data storage details without affecting the web services. Currently, our data sources include files on local volumes, tables in a PostGIS [8] database, objects in Amazon Simple Storage Service (S3) [9] and some external data sources hosted by collaborators.

### Benefits of the “Software as a Service” model

#### Easy access

Users can start using the applications without having to go through a software installation process. The applications can be easily accessed, especially for users who have to face the strict software installation policy often imposed by IT department of some corporations.

#### Automatic upgrade and maintenance

Because the software applications are centrally hosted, they can be easily upgraded by the service providers. All users use the same version of a software, which makes collaboration between users easier because there are no problems with software version mismatches. Bug fixes can be delivered quickly and easily. Users do not have to wait for the next release to get the bugs fixed.

#### Connectivity and resources sharing

In the Internet era, it is important that resources are connected and shared. The resources, such as computation, storage and software functionality, can be utilized more efficiently in a connected environment and users can collaborate more efficiently because data are connected and shared.

#### Platform independence

Users only need an Internet connection and a web browser to access the GPlates Portal. There is no significant platform restriction. The Cesium web applications harness modern technology to give users an interactive 3D experience. Users can rotate the globe, adjust the angle of view, change the height scale, and zoom into the very details of high resolution raster and terrain. Some portal applications are able to calculate data at a specified time/age according to the user input.

#### Cloud Hosting

The GPlates Portal is hosted in the cloud. Cloud hosting provides the following advantages, compared with traditional on-premises installation:

1. Scalability and flexibility. The traffic to our web services is highly variable. Spikes in the number of page requests coincide with the publication of datasets associated with high-impact journal articles, when the articles attract wide-spread media attention and general public interest. In light of the variable usage, it is important for the system to adjust its capability on-demand. The Cloud’s Auto Scaling service allows us to scale the system’s capacity up or down automatically according to the conditions. This auto scaling ability is important to ensure the quality of our web services without incurring a substantial infrastructure cost.
2. Lower cost and pay as you go. Using cloud hosting keeps the cost of hardware purchase and maintenance to a minimum. Furthermore, unneeded virtual machines in the Cloud can be terminated at any time, thus eliminating the issues related to disposal of computer hardware.
3. Launch virtual machine at designated locations. There is an obvious pattern in the distribution of user locations, which are mostly within Australasia, Europe and the USA. The Cloud allows us to launch virtual machines in these designated regions to improve the speed of network access.

## Free Software for Globe Design

One of the basic principles of the scientific method is that research results should be reproducible and independently verifiable [10] and one of the key components of reproducibility is open software. Free software is used in the development of the GPlates Portal, which gives readers easy access to the tools required to repeat the method described in this paper. The 3D rendering engine, Cesium Version 1.7.1 (http://cesiumjs.org/downloads.html), is released under the Apache 2.0 license. The reconstruction engine, GPlates/PyGPlates Version 1.5 (http://gplates.org/download.html), is released under GPLv2. The auxiliary Python programs in S1 Archive are released under GPLv2 in accordance with GPlates. The matplotlib Python library version 1.5.1 (http://matplotlib.org/1.5.1/index.html) is released under the Python Software Foundation (PSF) license.

## Virtual Globe Design and Construction

### Introduction

**Fig 2:**
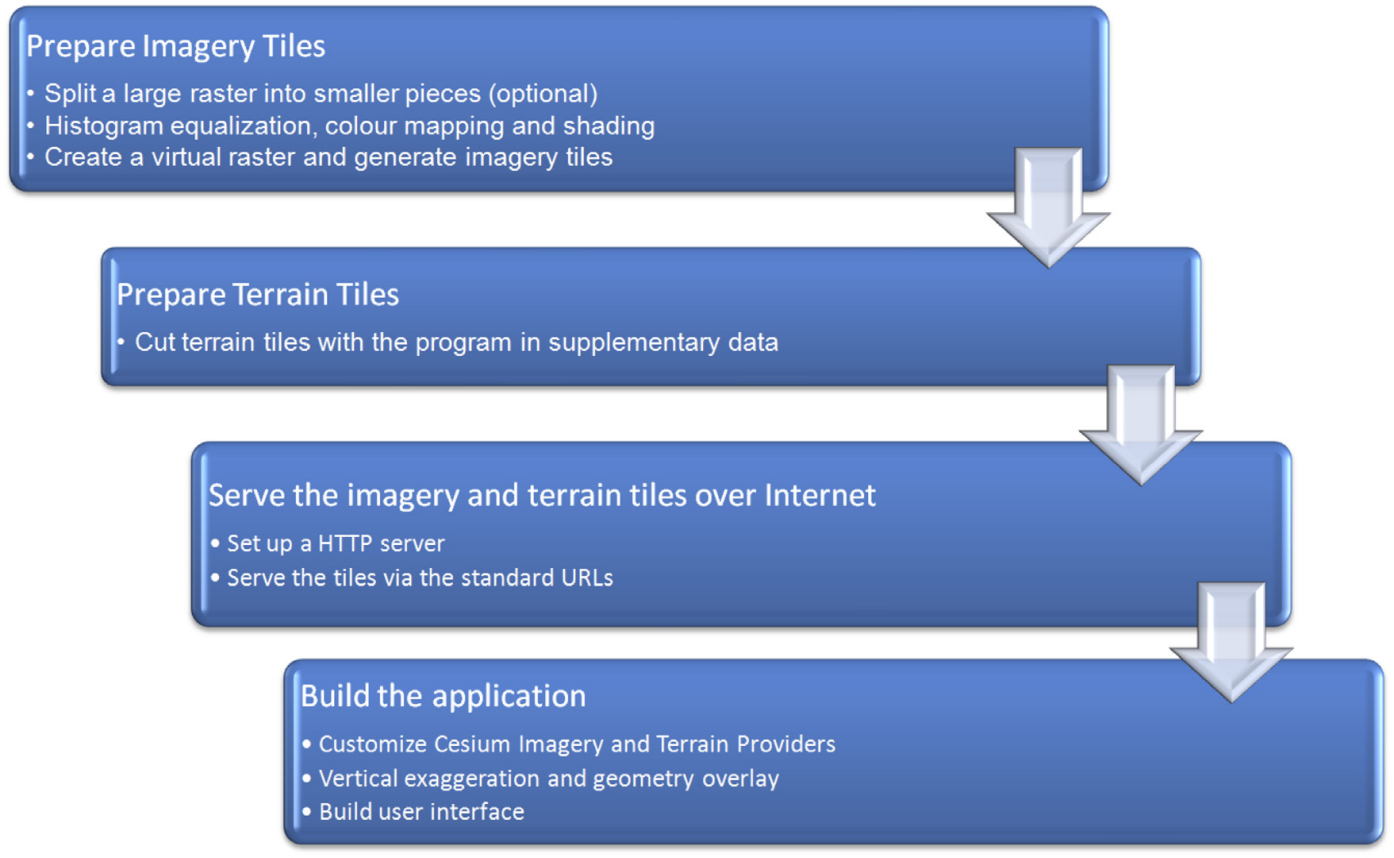
Workflow for designing a virtual globe. The details involved in this workflow are summarized in S1 Text.

Virtual globe design and construction consists of 4 major steps (Fig 2): (1) preparing imagery tiles, (2) preparing terrain tiles, (3) serving imagery and terrain tiles over the internet, and (4) building the application. The detailed methodology involved in design and construction, including python scripts, is available in S1 Text and supplementary python scripts are archived in S1 Archive.

### Design and functionality of individual virtual globes

In the following sections we describe a series of virtual globes that we have created as part of the GPlates Portal. Each globe is a companion to a published scientific research article. Indeed, each globe can be regarded as a powerful supplementary resource to these studies, allowing anyone with a web browser to visualize and interact with large data sets, reconstructions, and geodynamic simulation output that can only be partly represented within the limitations of a journal article.

### High-resolution topography and bathymetry

There are many Earth processes that can only be understood by mapping the topography of the deep oceans at high resolution. These include small-scale features such as abyssal hills, small seamounts (< 1 km tall), fractures that form on the outer walls of deep ocean trenches, and the erosional incisions on the seaward slopes of the continental margins. A new global grid of land topography and ocean bathymetry called SRTM15_PLUS (15 arc second resolution) was recently developed [11] following the methods of the SRTM30_PLUS grid [12]. The land topography is based on the SRTM3 mapping by the space shuttle in 2000 [13]. This is augmented by high-latitude data from ASTER stereo topography and a combination of CryoSAT-2 and IceSAT altimetry data for the ice caps of Greenland and Antarctica. The new aspect of the grid is the global bathymetry which is based on publically available depth soundings from a wide array of sources combined with the latest global bathymetric prediction from the global marine gravity grid discussed above [14]. In particular, high-resolution (100–200 m) multibeam echo sounder data from oceanographic institutions around the world [e.g., Lamont, Scripps, Woods Hole, JAMSTEC, GEOMAR, IFREMER, etc.] cover about 11% of the area of the oceans at 15 arcsecond resolution (~500 m). The older single-beam soundings from a global compilation maintained at the US National Geophysical Data Center provide 1 km spatial resolution over an additional 6% of the seafloor. Depths on the remaining 83% of the seafloor are interpolated from the global gravity grid [15]. This wide variety of data was assembled using the remove/restore method to bootstrap from the SRTM15_PLUS compilation. The assembly made extensive use of Generic Mapping Tools [16] for gridding as well as masking land/ocean areas. The SRTM15_PLUS grid will form the basis for the next version of Google Earth and is publicly available at ftp://topex.ucsd.edu/pub/srtm15_plus/.

**Fig 3:**
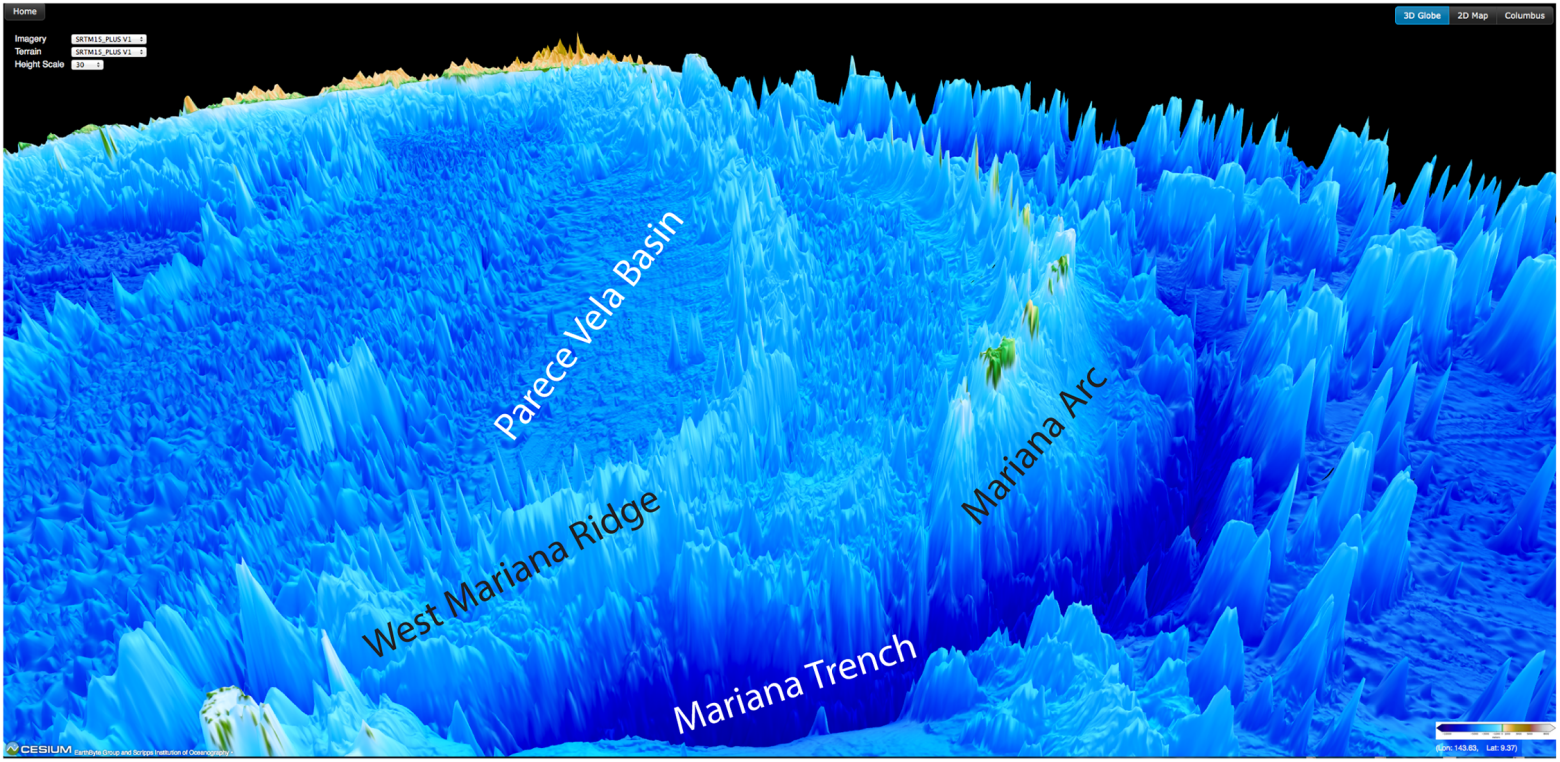
SRTM15 PLUS Cesium globe northward view of the region around the Mariana Arc and Trench. Recently added multibeam bathymetry data have substantially increased resolution of small-scale features in this region.

While all of the source data are publicly available and have been analyzed, the global assembly provides a new perspective on several types of ocean features. First, the spatial variations in abyssal hill fabric, not well resolved at 30 arc seconds, are now visible along most of the seafloor spreading ridge axes as well as some well surveyed areas with thin sediment cover. The amplitude and orientation of this abyssal fabric provides a window into the past spreading rate and direction. Significant new multibeam coverage from JAMSTEC in the Western Pacific provides remarkable resolution of the Izu-Bonin and Mariana trenches, Philippine Sea and Parece Vela Basin (Fig 3). A recent compilation of multibeam bathymetry from Geoscience Australia (http://www.ga.gov.au/scientific-topics/marine/bathymetry) provides vastly improved resolution of the continental margins and continental rise around Australia. New multibeam-based grids from GEOMAR reveal the detailed structure of the Central Chile Trench and outer trench wall.

### Seafloor lithology

**Fig 4:**
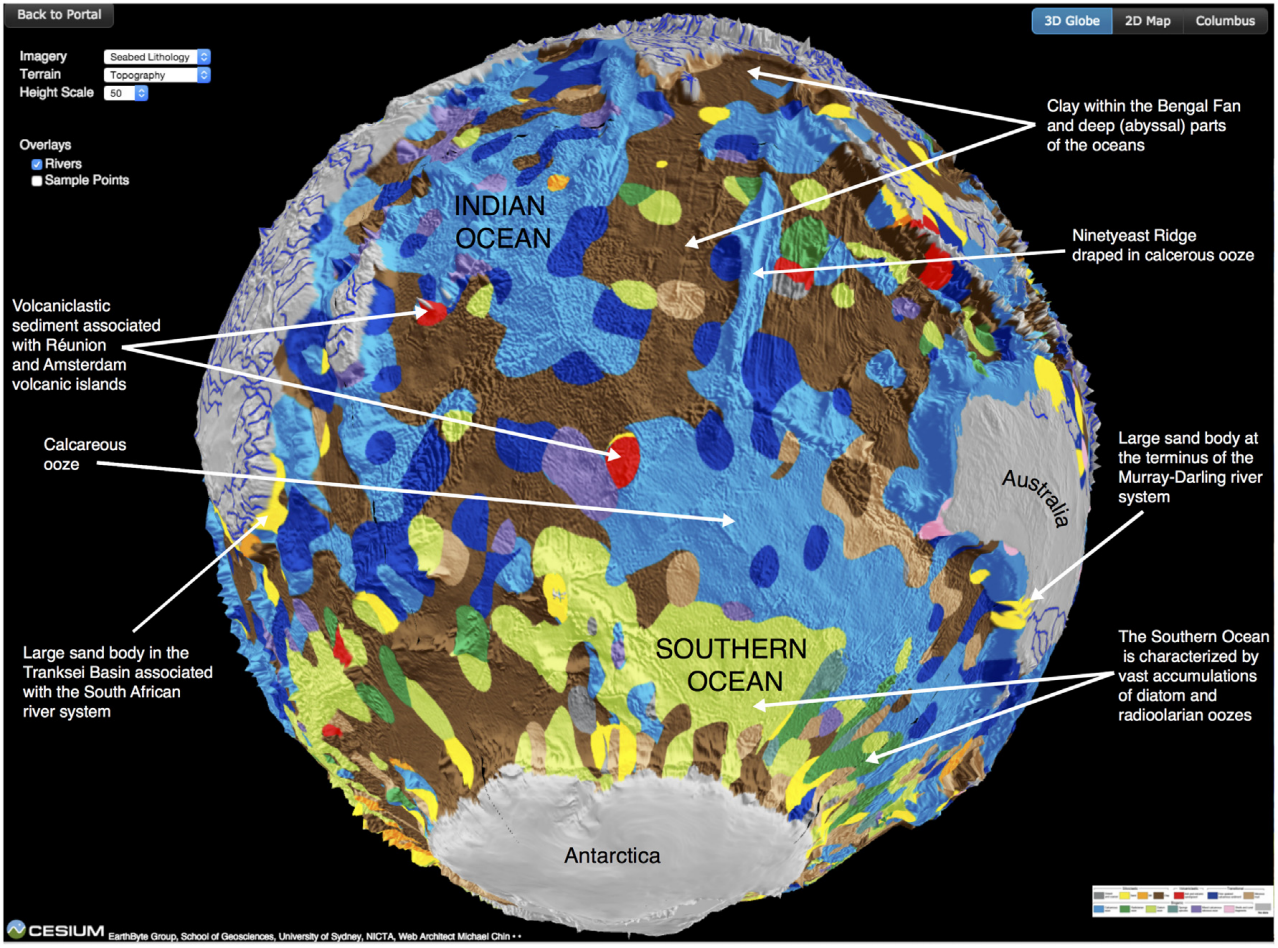
Cesium globe view of seafloor sediment types draped over a digital elevation model with an overlay of major rivers (blue lines). Several features of interest are highlighted.

The world’s ocean floor is draped by a vast blanket of sediments, which forms the largest geological deposit and the largest carbon sink on Earth (Fig 4). The composition of these sediments is highly variable and includes remains of plankton that have settled through the ocean column, terrigenous particles derived from continents and chemical precipitates that have formed in situ on the seafloor. The distribution of these sediments is critical for understanding how Earth has responded and continues to respond to environmental changes, the behavior of ocean currents, the occurrence of metal deposits and the assessment of geohazards. The recent publication of the first digital map of seafloor sediments based on the analysis and classification of nearly 15,000 surface sediment samples [17], provides a long overdue update on a highly generalized hand-drawn map of global seafloor sediments that dates back to 1974 [18] and features in virtually every textbook of marine geology and oceanography. The digital map was created using a bespoke machine-learning algorithm [17] and allows the joint analysis of these categorical data with oceanographic data that are freely available, for instance, through the National Oceanic and Atmospheric Administration (NOAA) (www.nodc.noaa.gov/OC5/WOD/pr_wod.html). Being open-access, the digital map is a valuable research resource for geologists, oceanographers and climatologists, and it has by far the highest viewing rate of the portal’s virtual globes at an average of ~500 views per day since August 2015. In its 3D visualization format that also features an optional overlay of world’s major rivers, it is a powerful and exciting teaching tool for all levels. As seafloor lithology is draped over a digital terrain map, the globe allows the viewer to explore how the distribution of different sediment types varies between ocean basins and with latitude, forming the basis for making connections with a variety of processes that govern the distribution of either biogenic or lithogenous sediments. For instance, the globe can be used to explore how the type of seafloor sediment depends on proximity to mid-ocean ridges, volcanic plateaus, different types of continental margins (tectonically passive or active), major river mouths and different climatic zones.

### Vertical gravity gradient

**Fig 5:**
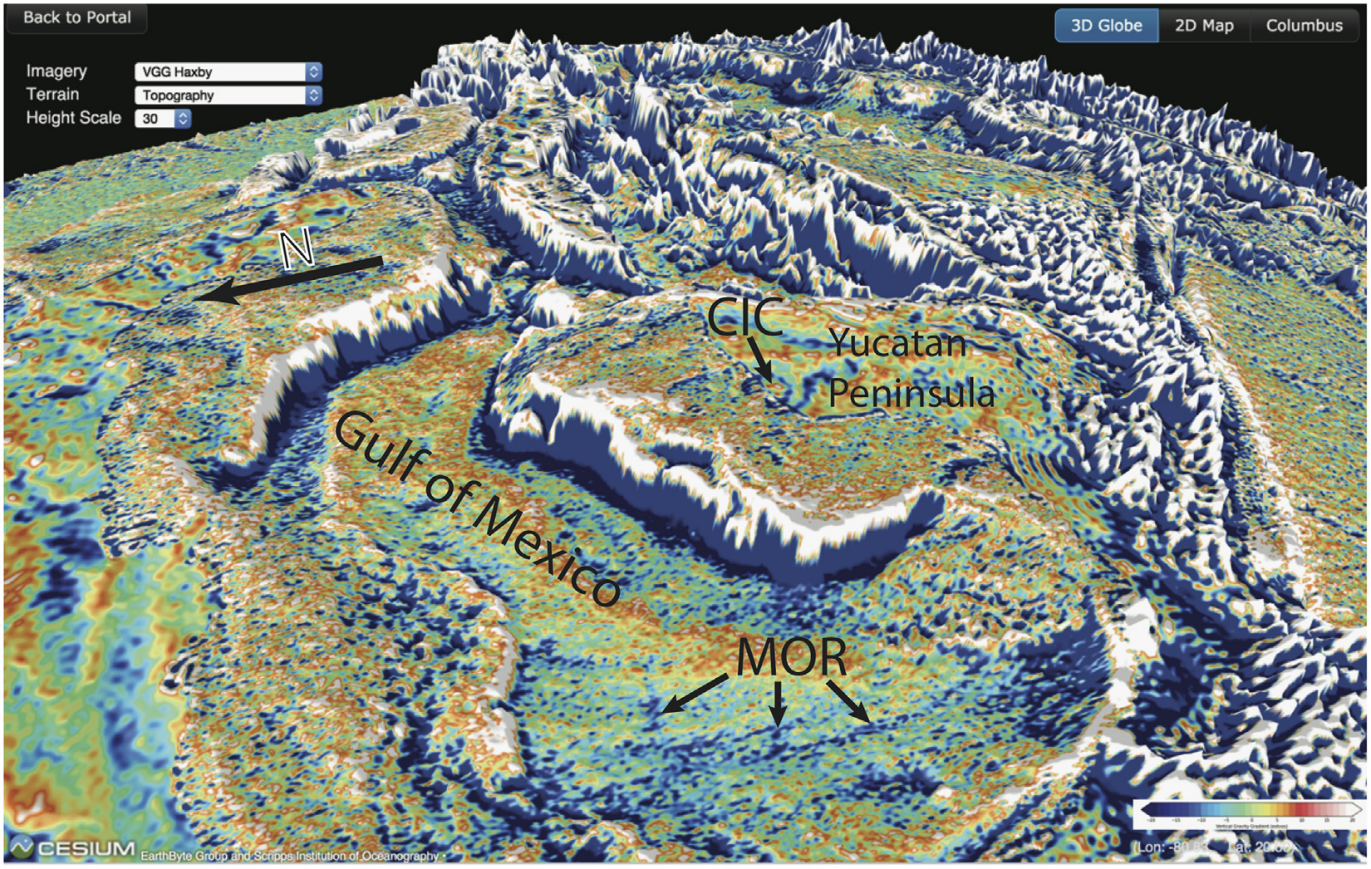
Cesium globe southeastward view of the Gulf of Mexico and the Yucatan Peninsula where the vertical gravity gradient grid, overlain by a digital elevation model, reveals the buried circular structure related to the Chicxulub Impact Crater (CIC) that led to the extinction of the dinosaurs and many other species, straddling the coastline of the Yucatan Peninsula [21]. Impact-generated multi-ring gravity anomalies can be used to compute the impact’s size and other characteristics [21]. The image also reveals the buried extinct Jurassic mid-ocean ridge (MOR) of the Gulf of Mexico [14].

Gravity models are powerful tools for mapping the structure of the ocean floor, especially in the deep ocean basins where the topography remains unmapped by ships or is buried by thick sediments. A recently published global marine gravity model [14], which is twice as accurate as previous models, has here been combined with the continental gravity model EGM2008 [19] to produce a global model for the free-air gravity field. The digital free-air gravity model was used to compute the vertical gravity gradient, which enhances small-scale gravity anomalies, related to tectonic structures such as small seamounts and faults, horst and graben structures at the seabed or buried underneath the seafloor. The vertical gravity gradient is particularly useful for visualizing deep structures associated with continental margins and sedimentary basins, where seismic imaging data are sparse and expensive to obtain. The vertical gravity gradient dataset was used to create the first of the GPlates Portal virtual Cesium globes in October 2014; it has since been viewed nearly 100,000 times *(31*^*st*^ *January 2016)*. While thousands of meters of water obscure our view of the array of geological features characterizing the seabed and buried sub-seafloor geology, the marine portion of this globe reveals many previously unknown seafloor and sub-seafloor structures, including an extinct mid-ocean ridge from the Jurassic Period in the Gulf of Mexico (now buried by up to 8 km of sediments) and a huge ancient rift scar in the South Atlantic Ocean formed by a deep-seated “hotspot” leading to a cracking of the ocean floor [14]. By providing a seamless view of the combined marine and continental gravity field, this Cesium globe allows the user to follow structures from the deep ocean basins to continental slopes and shelves to the continents themselves. Such structures may include oceanic fracture zones, many of which have an origin in tectonic lineaments in the continental crust which existed before continental breakup [20], as well as meteorite impact structures (Fig 5).

### Global magnetic anomalies

Ferrous minerals in the crust and upper mantle give rise to magnetic anomalies. The strength and shape of these anomalies reflects the mineralogy and thickness of geological formations. Since minerals become non-magnetic above their Curie temperature, magnetization has a limited depth extent, which depends on the local temperature gradient of the lithosphere. Magnetic anomalies therefore provide insight into subsurface structure, composition and heat flow in the Earth's crust. Anomalies trending parallel to the isochrons (lines of equal age) in the oceans reveal the formation (seafloor spreading) and destruction (subduction zones) of oceanic crust, the formation of continental crust by accretion of various terranes to cratonic areas and large-scale volcanism on continents and in the oceans.

**Fig 6:**
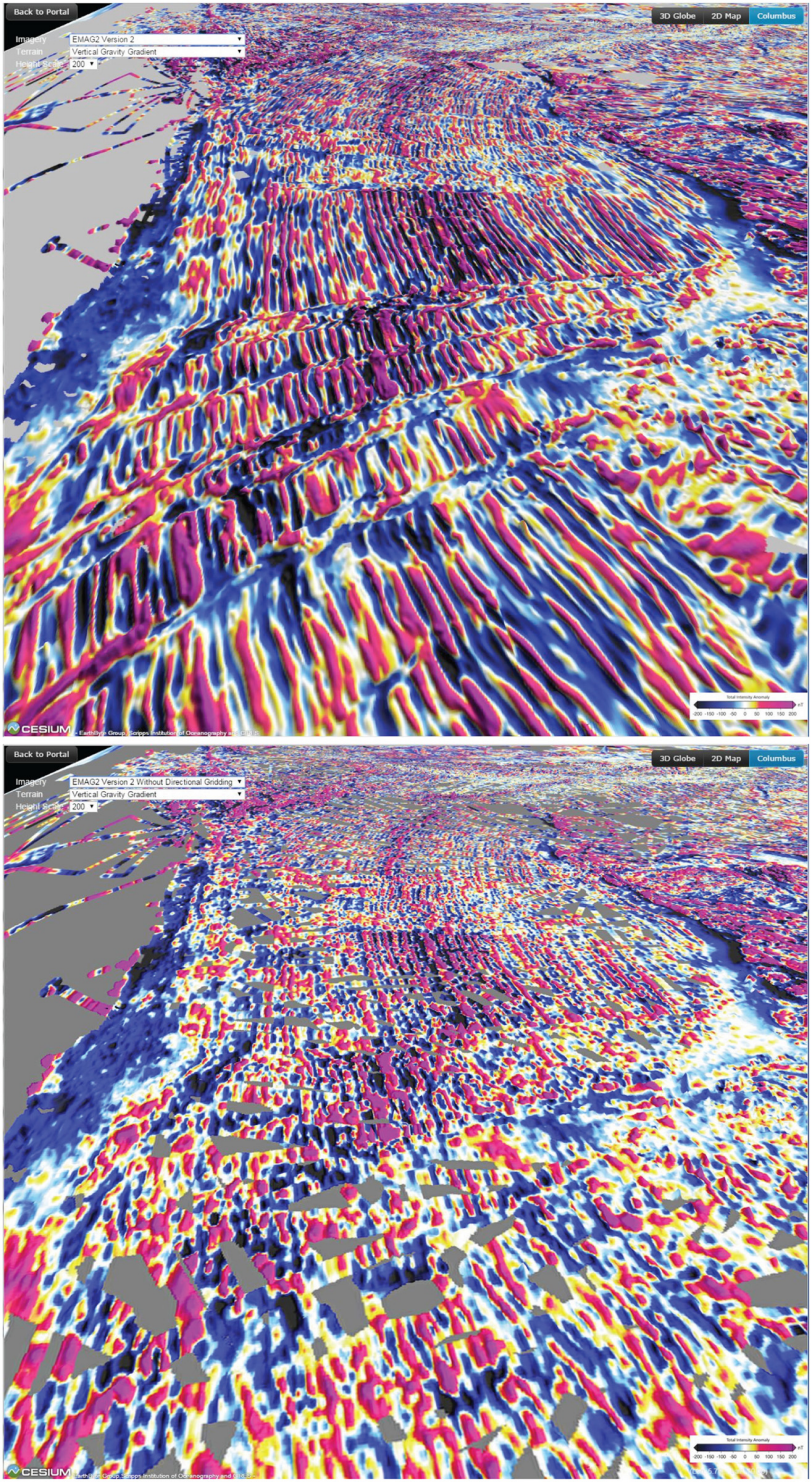
Identical view illustrating the difference between the EMAG2 version with directional gridding (top) and with isotropic gridding (bottom). The observer location is south of New Zealand looking westward at the Southern Ocean, with Antarctica on the southern side and Australia on northern side in each image.

Magnetic anomaly data have been collected by ships, aircraft, and low-orbiting satellites for more than half a century, providing global coverage of the Earth. The Earth Magnetic Anomaly Grid in 2 arc minute resolution (EMAG2) is the result of an international collaboration based on contributions from over one hundred data providers worldwide [22]. The input data consisted of both 2D grids and 1D profile data from ship and airborne measurements. These were processed, line levelled and merged at 4 km altitude using least squares collocation. Two versions of the EMAG2 grid were produced. One with conventional isotropic gridding and one with anisotropic (directional) gridding over the oceans, in which the anomalies were “stretched out” in the direction of the local isochrons as given by the oceanic crustal age model [23]. A comparison of EMAG2 with and without directional gridding is shown in Fig 6. Finally, contributions with spatial wavelengths larger than 330 km were replaced with the CHAMP satellite magnetic anomaly model MF6 [24].

### Global magnetic anomaly identifications

**Fig 7:**
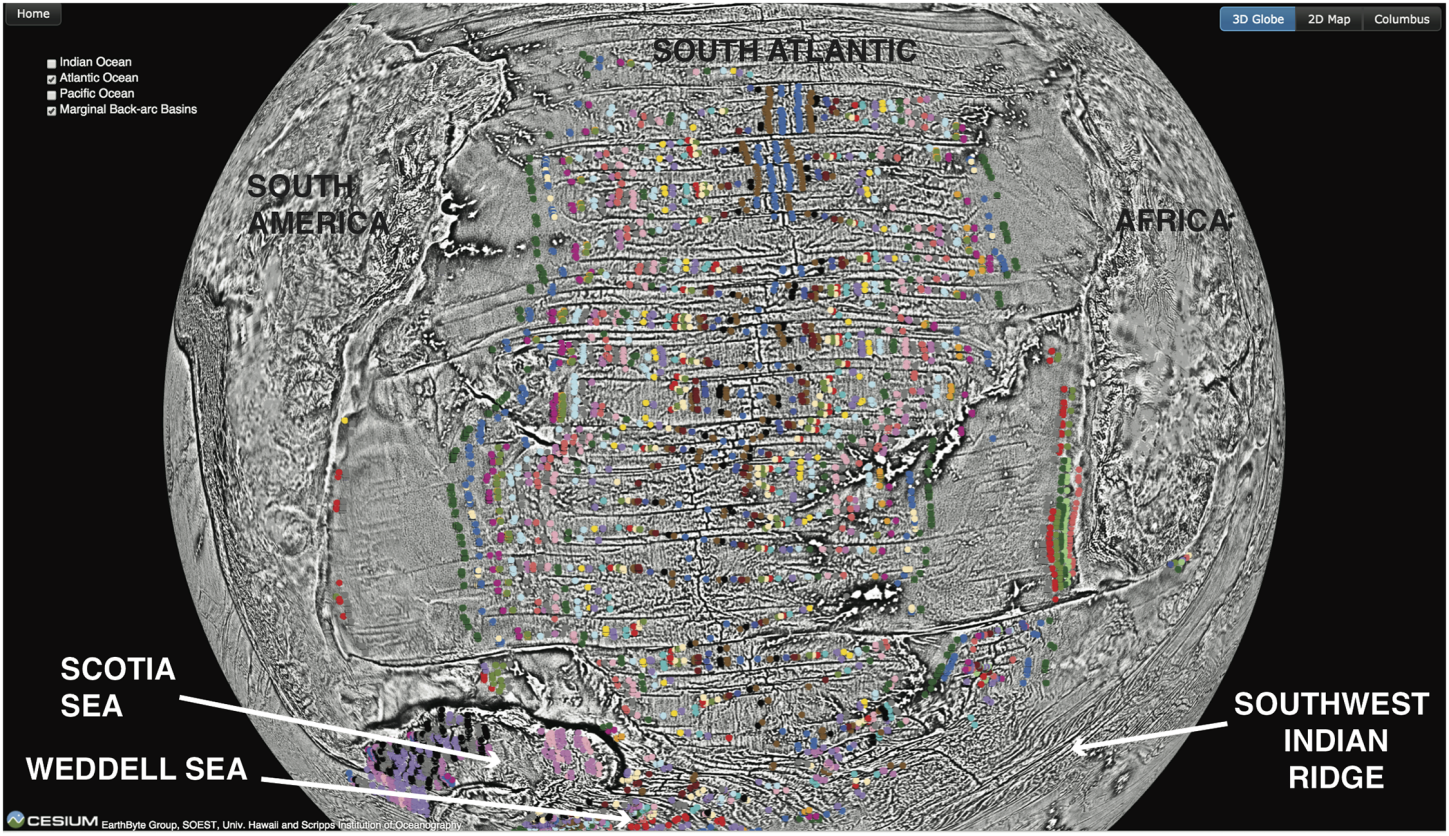
Cesium globe of the magnetic anomaly identification data [25] focussed on the South Atlantic. Colored circles represent individual magnetic anomaly identifications color-coded by magnetic chron name. Seafloor-spreading between Africa and South America is represented by the magnetic anomaly picks in the South Atlantic, between Africa and Antarctica along the Southwest Indian Ridge, between South America and Antarctica in the Weddell Sea and several episodes of back-arc basin opening in the Scotia Sea.

Marine magnetic anomaly identifications are one of the primary building-blocks of global plate reconstructions, used to determine the age of the oceanic crust and to understand past seafloor-spreading processes. These magnetic anomaly identifications, often called “magnetic picks”, are a spatio-temporal representation of interpreted marine magnetic anomalies described above against a geomagnetic reversal timescale. In the past, most global and regional compilations have been presented as a series of maps with no accompanying digital data. An open-source, community-driven infrastructure containing over 96,000 published magnetic anomaly identifications was recently established [25]. The repository contains consistently described and well-documented data points that have undergone internal quality-control. While all the data have been made accessible to the public via a dedicated website (www.soest.hawaii.edu/PT/GSFML), the repository lacks a visualization tool for expert and non-expert users to view and interrogate the data. These data can be visualized in the Cesium globe at the present day (Fig 7), color-coded by magnetic chron end, which allows users to get a sense of global data coverage, to further understand the parts of the global plate motion model that are underpinned by a robust set of magnetic anomaly identifications, and to explore seafloor-spreading regimes. In the future, tools to interrogate the data attributes will be made available.

### Gravity and magnetic grid reconstruction through geological time

Since geophysical signatures of the oceans and continents contain information about past plate configurations, a natural step is to reconstruct these data using models for past plate configurations, both to validate existing models and explore alternative scenarios. The GPlates desktop software provides functionality to perform high-resolution raster reconstructions [26]. Different portions of geo-referenced images or gridded geophysical data are assigned to tectonic plates linked to a global rotation model that describes how all major plates have moved relative to each other, and relative to the Earth’s spin axis, since 200 million years ago [27]. The workflow is straightforward for experienced GPlates users, but barriers exist to the wide use of such technologies—users need to download and install GPlates software, access and store large volumes of high-resolution image data, and follow tutorials describing how to use the data and software together.

**Fig 8:**
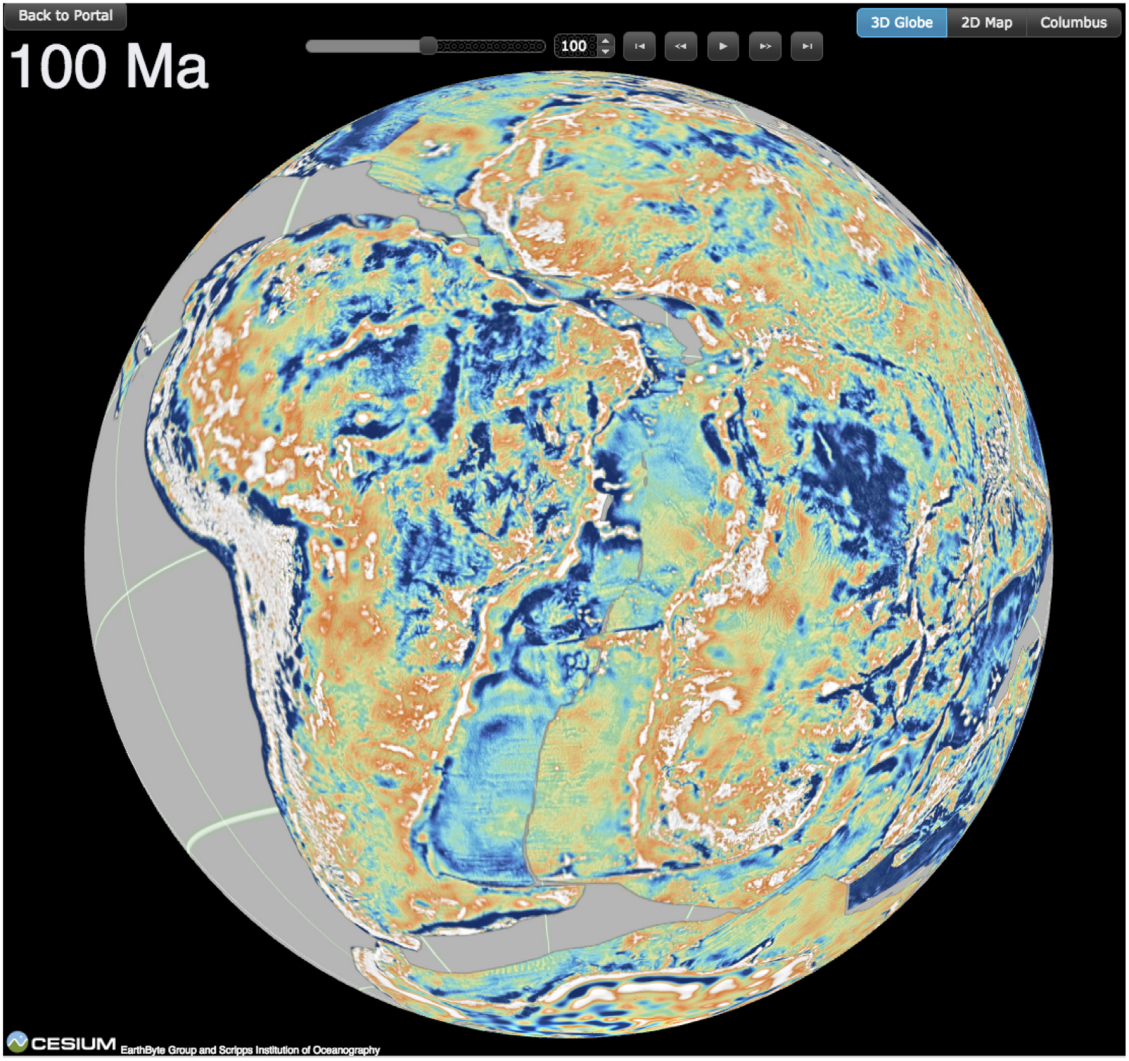
Cesium globe illustrating a reconstruction of the combined marine and continental gravity grid at 100 million years ago, centered on the western Gondwana continents, including South America on the left and Africa on the right.

The GPlates Portal currently contains globes in which the free-air gravity grid [14] (Fig 8) as well as the magnetic anomaly grid EMAG-2 [22] can be reconstructed back through geological time on separate Cesium globes. High-resolution image data are linked to a plate tectonic reconstruction using GPlates desktop software, then exported and tiled as a time-dependent raster sequence. A set of controls added to the Cesium globe interface enables the sequence to be played backwards and forwards through geological time. These globes allow users to explore how now widely separated tectonic structures, as expressed in their gravity and magnetic anomaly signatures, were once juxtaposed when the supercontinent Pangea existed 200 million years ago.

### Surface dynamic topography through time

Convective motions within the Earth’s mantle result in deformation of its surface that is often referred to as “dynamic topography”. We recently developed a method to use global tectonic reconstructions as boundary conditions of mantle flow models [28], which allows us to model the evolution of long-wavelength dynamic topography deeper in geological time than other methods [29]. We derive air-loaded dynamic topography *h* at the Earth’s surface resulting from mantle flow deeper than 350 km. The calculations use either free-slip or no-slip boundary conditions, and the reader is referred to the publications associated with the models available on the GPlates Portal for further details of each calculation of dynamic topography.

**Fig 9:**
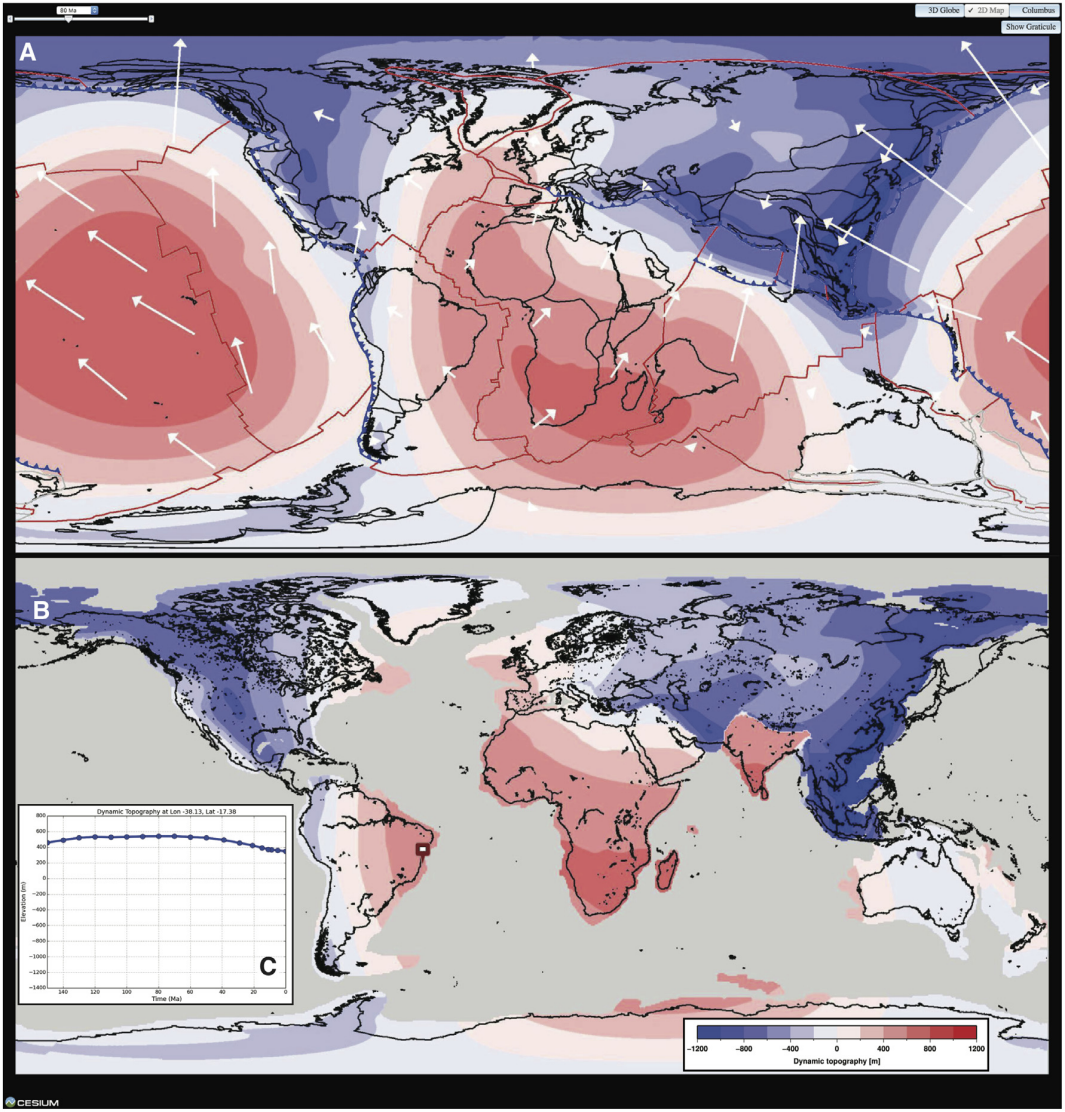
A. 2D-map view of global dynamic topography in the mantle frame of reference at 80 Ma for case 1 of ref. 31. Reconstructed coastlines are shown in black, subduction zones in blue (triangles indicate dip direction), mid-ocean ridges and transform faults in red, deforming areas in grey, and surface velocities as white vectors. B. 2D-map view of global dynamic topography in the plate frame of reference at 80 Ma, with present-day coastlines in black. C. Evolution of dynamic topography along the East Brazil Rift System (dark-red rectangle in B).

The results of our global models show that the interplay between the motion of tectonic plates and the changing shape of the Earth due to large-scale mantle convection results in topography variations of less than ±2 km, evolving over a time scale of ~ 10 Myr. Results are presented in the reference frame of the mantle (Fig 9A), which captures the evolution of global dynamic topography, as well as in the reference frame of tectonic plates (Fig 9B), to ease direct comparison with geological observations [30, 31]. In the plate frame of reference, the evolution of dynamic topography at a given point can be plotted (Fig 9C) and downloaded in csv format. Current models extend back to 230 Ma and do not include active mantle upwellings [31, 32]. However, we are currently working on models which extend further back in geological time and include active mantle upwellings, and they will eventually be added to the GPlates Portal.

## Discussion

### Complementary roles of web-based versus desktop virtual globes

The difficulties in developing interactive browser-based tools to substitute a given desktop application used to be prohibitive until recently, certainly for scientists outside of major national research institutions. However, now a new generation of open-source tools is facilitating a “virtual globe revolution” by allowing researchers anywhere to participate in the construction of online virtual globes. The GPlates Portal is an example of this revolution and represents a new way to visualize and share geophysical and geological data. Compared to traditional desktop software applications, the main advantage of the GPlates Portal web applications is the software distribution model Software as a Service (SaaS). In the SaaS model, the software applications are hosted by service providers and made available to users via the Internet and the access to services is usually controlled by user subscriptions.

Web-based applications such as the GPlates Portal still have limitations compared to more traditional applications. For example, the GPlates desktop software contains a range of tools for ‘power-users’ to perform complex tasks such as interactively modifying reconstructions, visualizing evolving plate boundaries geometries and plate kinematic indicators. Applications within the GPlates Portal are deliberately free of such complications, and are designed to complement desktop applications rather than replace them—they provide a more streamlined, user-friendly interface to explore the same data sets and reconstruction models used in desktop applications. Traditionally, scientists have shared plate reconstructions through animations, or more recently through video-sharing sites such as Youtube (www.youtube.com/channel/UCa41IQEhmmuXmz9J6iMfsnA). Such animations are inherently limited since they are not interactive—GPlates portal applications perform a similar function to such animations, but are vastly more powerful, providing an immersive environment for users to interact with scientific datasets.

### Future Extensions

The GPlates Portal has been designed as a gateway to a knowledge and service hub for geologists and geophysicists interested in understanding Earth’s surface and crustal structure and Earth evolution through geological time. Any researchers investigating spatial data sets can utilise the technologies described here to generate their own virtual globes of raster, vector or point data, overlain over a digital terrain model, based on the open software underpinning globe design. Our experience shows that raster data are best suited for fast interactive globe visualization, while large volumes of vector or point data overlays tend to make interactive globe manipulation more sluggish. Future opportunities for extension of virtual globe functionality include allowing users to submit their own data to create customized Cesium applications. Customized Cesium applications may be created automatically based on the data provided by users. In this way, even users with little knowledge about web application development can publish and visualize their data interactively in the Cloud. Future functionality will also include making big data mining workflows accessible as services on the Cloud. Finally, the establishment of RESTful web services will migrate some functionality of the desktop GPlates software [33] into the Cloud. The portal already includes a prototype for a paleomapmaker, designed to allow users to export publication-quality figures using datasets and reconstructions contained within the portal data store or uploaded directly by users.

## Conclusions

The GPlates Portal provides a series of virtual globes that put fast interactive visualization of global geophysical and geological data sets and paleomap-making into the hands of public end-users. The portal has been visited nearly 300,000 times since its inception in October 2015, as tracked by google analytics, and the globes have been featured in numerous media articles around the world. They have helped illustrate online articles on published research papers by media either providing links to a given virtual globe or by embedding a globe in their own web page. In this way the globes have started playing a significant role in helping to communicate research to the public. Their popularity demonstrates the demand for fast visualization of global spatial big data, both for the present-day as well as through geological time. The cloud-based Cesium globes offer many future opportunities for providing additional functionality, especially on-the-fly big data analytics.

## Supporting Information

S1 Archive Supplementary Python Scripts. These scripts include color mapping to raster data, applying histogram equilization to raster data, applying shading to raster data, splitting a large grid into a set of smaller grids, and generating terrain tiles from an elevation grid.
(ZIP)

S1 Text Virtual Globe Design Methodology. This includes the preparation of imagery tiles, terrain tiles, serving imagery and terrain tiles via URL, and building portal applications.
(PDF)
