# Supplementary material for: The GPlates Portal: Cloud-Based Interactive 3D Visualization of Global Geophysical and Geological Data in a Web Browser
Source: PLoS One. 2016 Mar 9;11(3):e0150883. doi: 10.1371/journal.pone.0150883 (PMC4784813; doi:10.1371/journal.pone.0150883)
Supplement: S1 Text — This includes the preparation of imagery tiles, terrain tiles, serving imagery and terrain tiles via URL, and building portal applications. (PDF) [file pone.0150883.s002.pdf]

The GPlates Portal: Cloud-based interactive 3D visualization of global geophysical and geological data in a web browser

R. Dietmar Müller, Xiaodong Qin, David T. Sandwell, Adriana Dutkiewicz, Simon E. Williams, Nicolas Flament, Stefan Maus and Maria Seton

S1 Text

Virtual Globe Design Methodology

1) ***Preparing imagery tiles***

a) Splitting large rasters into smaller pieces

Usually, the size of high-resolution raster files is very large, for instance, the size of the SRTM15_PLUS global digital elevation model (ftp://topex.ucsd.edu/pub/srtm15_plus) is more than 15 gigabytes. It is very difficult, if not impossible, to process such large raster files on desktop or laptop computers because of hardware and architecture limitations. Thus, we split the large raster into smaller-sized pieces for further processing. The following GDAL command:

"gdal_translate –projwin 0 0 10 10 input.tif output.tif"

copies the raster data in a bounding box from the georeferenced input raster into an output file. The rectangular bounding box is defined by the upper left corner coordinates (0, 0) and lower right corner coordinates (10, 10). We supply the Python

script split_grid.py (S2 Script) which calls gdal_translate repeatedly to cut a large raster file into smaller pieces.

b) Histogram equalization, color mapping and shading

Before applying simple linear or injective color mapping, histogram equalization on raster data is necessary in order to avoid the dominance of a narrow range of the color spectrum to be applied to the image. The color mapping function can be a simple linear transformation, injective function or a more complicated image processing algorithm. ImageMagick's 'Pegtop_Light' composition method and Esri's hillshade algorithm are used for shading in the GPlates Portal. We supply three Python scripts (S3-S5 Scripts) as examples of histogram equalization, color mapping and shading. The histeq.py contains a function which applies histogram equalization and normalization to a two-dimensional numpy array. The shade() function in shading.py applies shading to grid data. And the colouring.py constructs a matplotlib colormap object from a list of colours and values, then applies color mapping to input data.

c) Generate imagery tiles

Before generating imagery tiles for Cesium, all the georeferenced image pieces must be bound together by creating a virtual raster with GDAL. The following GDAL command:

*gdalbuildvrt example.vrt *.tif*

creates a virtual raster from all the georeferenced ".tif" files in the current working directory. Eight levels of imagery tiles are then created for the input raster using the command

The dimensions of tiles created are 256*256:

gdal2tiles.py –zoom='0-8' –s EPSG:4326 inputfile outputdir

The coordinates of imagery tiles comply with the OSGeo Tile Map Service Specification. Figure 3 illustrates the tile coordinate system. The number of tiles is 2^{n+1} at level n. The number of tiles increases exponentially with zoom level. Cesium uses this tiled imagery mechanism to display the very details of high resolution rasters.

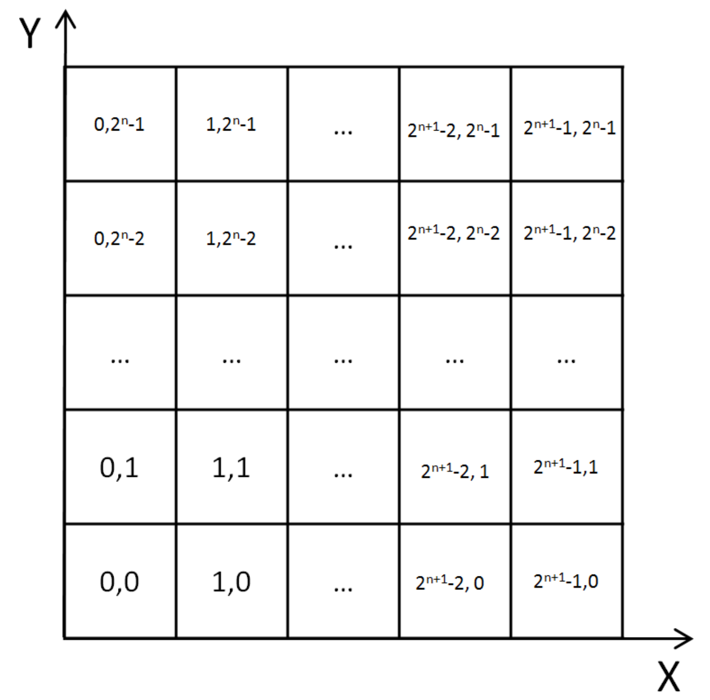

Fig 1. Diagram of tile coordinates

Fig 1. shows an example of 4 tiles at zoom level 6 from the global Vertical Gravity Gradient (VGG) map. The tiles are stored in separate files and Cesium will seamlessly join the tiles and render the image on the 3D globe.

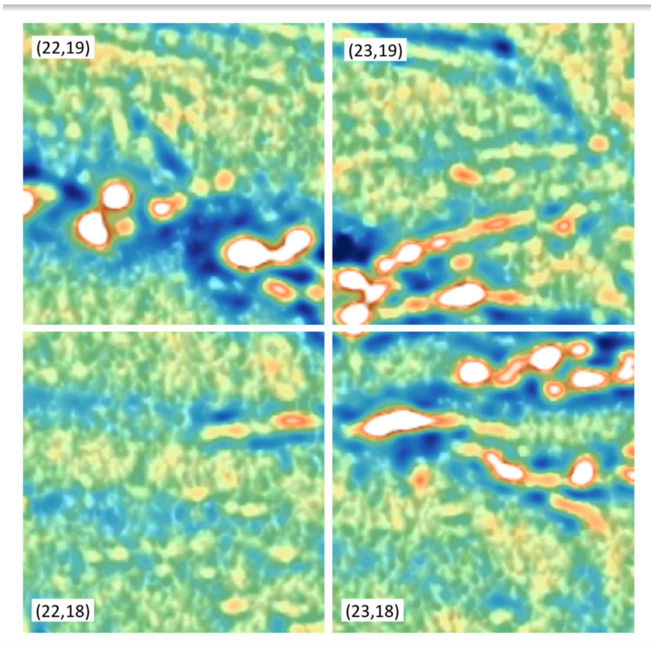

Fig 2. Example tiles from the global Vertical Gravity Gradient (VGG) map

2) *Preparing terrain tiles*

Terrain data are also known as a Digital Elevation Model (DEM). Cesium supports two terrain data formats -- heightmap-1.0 and quantized-mesh-1.0. The coordinates of terrain tiles are defined in the same way as imagery tiles. Each terrain tile has 65*65 height data and overlaps its neighbours at the edges. The terrain tiles are compressed in gzip format in order to save disk space and minimize network traffic. The size of an extracted tile file should be at least 8,452 bytes. The tile data consists of three parts.

1. Height data

Each height value is a 2-byte integer (int16). In total, the height data are 65 * 65 * 2 = 8450 bytes.

2. Child Mask

Each terrain tile can have up to 4 child tiles. An 8-bit child mask follows the height data immediately, which indicates the presence of child tiles. Only the last 4 bits are used.

3. Water Mask

There is no need to render water in our applications. Thus, a 1-byte water mask is used and the value is set to 0 to indicate that all terrain should be rendered as land.

We supply the Python script terrain_tiles.py (S6 Script) as an example of generating terrain tiles from elevation data.

3) *Serving imagery and terrain tiles via URL*

All the imagery and terrain tiles must be accessible via URLs. A HTTP server is required to serve tiles via HTTP protocol. The URLs must follow the standard convention. The standard URL convention is as follows:

http://YOUR-ROOT-URL/zoom-level/x/y.[jpg | terrain]

The “YOUR-ROOT-URL” is the root location chosen to host the tiles. The “zoom-level” indicates the level number of the tiles. The “x” and “y” are the tile coordinates. The extension of imagery tiles is “.jpg”, and for terrain tiles, it is “.terrain”.

4) Building applications

a) Creating customized Imagery Provider and Terrain Provider for Cesium

   Two interfaces must be implemented to create customized Imagery Provider and Terrain Provider.

   i) The ImageryProvider Interface

   ii) The function requestImage() must be overridden in the customized imagery provider. Cesium calls this function to get imagery tiles at runtime. requestImage() takes the zoom level and tile coordinates as input and return the requested imagery tile.

   iii) The TerrainProvider Interface

      The most important function which must be overridden in customized terrain provider is requestTileGeometry() which takes zoom level and tile coordinates as input and returns terrain tile data.

b) Vertical exaggeration

      Most topography on Earth is too insignificant to be noticeable at the global scale without vertical exaggeration. Therefore, vertical exaggeration is required to visualise terrain effectively. Vertical exaggeration can be achieved easily by multiplying the elevation data by a factor in the requestTileGeometry() function. In the GPlates Portal, this factor can easily be controlled by users via the “Height Scale” dropdown list.

c) Geometry overlay

Vector data can be drawn on the Cesium globe and maps as polygons, polylines and points. The geometry overlay allows users to mark the geological features on the globe.

d) User Interface

The jQuery library can be used to create GUI elements to control the main canvas or display additional information.
